# Resina Draconis Promotes Diabetic Wound Healing by Regulating the AGE-RAGE Pathway to Modulate Macrophage Polarization

**DOI:** 10.3390/cimb47090748

**Published:** 2025-09-11

**Authors:** Xin Jin, Ang Li, Zhaoyuan Dai, Yi Li, Xinchi Feng, Feng Qiu

**Affiliations:** School of Chinese Materia Medica, Tianjin Key Laboratory of Therapeutic Substance of Traditional Chinese Medicine, Tianjin University of Traditional Chinese Medicine, 10 Poyang Lake Road, West Zone of Tuanbo New City, Jinghai District, Tianjin 301617, China; jinxin041@163.com (X.J.); lianghbkd@163.com (A.L.); zhaoyuandai1024@163.com (Z.D.); 15369620697@163.com (Y.L.)

**Keywords:** Resina Draconis, diabetic wound, AGE-RAGE, macrophage polarization

## Abstract

Resina Draconis (RD), a traditional Chinese medicine, has been widely used in treating diabetic foot ulcers. However, its specific mechanisms of action remain incompletely understood. First, network pharmacology combined with GEO clinical sample data mining was employed to systematically analyze the therapeutic targets of RD in promoting diabetic wound healing. Second, an AGEs-induced RAW264.7 cell model was utilized to investigate the regulatory effects of RD and its primary active components on the AGE-RAGE signaling pathway, along with their anti-inflammatory and antioxidant activities. Finally, a diabetic wound mouse model was established to validate the efficacy of RD and further explore its underlying molecular mechanisms. Integrated analysis of network pharmacology and GEO database mining identified 492 potential therapeutic targets of RD in diabetic wound healing, primarily involving the AGE-RAGE pathway. In vitro, RD (6.25 μg/mL) significantly suppressed AGE-induced inflammatory factors and ROS production while downregulating AGE-triggered RAGE protein overexpression. In vivo, RD hydrogel accelerated diabetic wound healing by modulating the AGE-RAGE axis and regulating macrophage polarization. RD effectively promotes diabetic wound healing through synergistic regulation of the AGE-RAGE pathway, oxidative stress suppression, and macrophage polarization modulation, providing a novel therapeutic strategy for diabetic wound management.

## 1. Introduction

Globally, diabetes mellitus (DM), a serious public health concern, is growing at a startling rate. Diabetic foot and other diabetic vascular problems are primary contributors to disability and mortality in diabetic patients [1]. An amputation for a diabetic patient occurs every 30 s worldwide, according to statistics [2]. In addition to having a complicated etiology and being impacted by a wide range of therapeutic parameters, diabetic chronic wounds are also likely to recur and have an unknown prognosis, which places a significant economic burden on the patient. Diabetic foot issues can make up as much as 40% of diabetes-related healthcare expenses in developing nations, compared to 12–15% in industrialized nations [3]. Four sequential phases make up the organized biological process of wound healing: hemostasis, inflammation, proliferation, and tissue remodeling. Delays in wound healing may result from any deviation in these phases [4,5]. Advanced glycation end products (AGEs) are created and accumulate in diabetes patients as a result of aberrant biochemical metabolism, especially persistent hyperglycemia, which encourages the interaction between free amino groups on proteins and carbonyl groups on carbohydrate [6,7]. The receptor for advanced glycation end products (RAGE) is one of the main targets of AGE signaling, and the AGE-RAGE axis is important in the complications associated with diabetes [8]. RAGE is a transmembrane protein with a distinct structure that functions as a pattern recognition receptor. Along with a transmembrane domain and an intracellular segment in charge of signal transduction, its extracellular region consists of two constant domains (C1 and C2) and a variable domain (V domain) [9,10]. RAGE’s particular binding to AGEs in diabetic wounds activates NF-κB, which in turn promotes the generation of pro-inflammatory cytokines, including TNF-α [11], keeping tissues and cells in a chronic inflammatory state and impeding wound healing. Furthermore, AGEs’ particular affinity to RAGE increases the production of ROS, which exacerbates oxidative stress and inflammation [12].

RAGE signaling is notable for its positive feedback properties. Through transcription factors like NF-κB, it upregulates its expression upon activation, resulting in a vicious cycle of oxidative stress and inflammation [13]. Under physiological settings, macrophages can sense microenvironmental cues and dynamically switch from the early pro-inflammatory M1 phenotype to the late anti-inflammatory M2 phenotype. This mechanism directly upsets the balance of macrophage polarization. The healing of diabetic chronic wounds is significantly hampered by persistent AGE-RAGE signaling, which “freezes” macrophages in the M1 state and continuously attracts and activates new M1 macrophages through positive feedback. This creates a chronic inflammatory microenvironment that is hard to reverse [14,15].

Diabetic wounds present many difficulties for current therapeutic approaches. Although glycation crosslink breakers like aminoguanidine (AG) and N-phenacylthiazolium bromide (PTB) mostly prevent the production of AGEs, they have serious side effects, high development costs, and long-term drug requirements [9]. Growth factors, including basic fibroblast growth factor (bFGF) and epidermal growth factor (EGF), can aid in wound healing, but their use is restricted by their high expense, short half-lives, and vulnerability to inactivation [16]. Even while blood sugar can be controlled by insulin, SGLT-2 inhibitors, and GLP-1 receptor agonists [17,18], irreversible microvascular/neuropathic damage may cause wounds to not heal. Additional therapies include local antibiotic therapy [19] and cell therapy [20]; however, these approaches are frequently costly and provide erratic outcomes. Finding clinically viable medications to treat diabetic foot sores has thus become a pressing concern.

Resina Draconis, derived from the resin extracted from the lipid-containing wood of the lily family plant *Dracaena cochinchinensis* (Lour.) S.C.Chen, is also known as dragon’s blood [21]. It has been widely used in traditional medicine in Arab, Chinese, Thai, and other regions [22]. Modern pharmacological studies have shown that Resina Draconis promotes skin repair, exhibits anti-inflammatory and antibacterial effects, reduces blood sugar and lipids, shows anti-tumor properties, and inhibits platelet aggregation [21,23]. Notably, it has shown promising efficacy in treating refractory wounds at a lower cost compared to the aforementioned drugs [24]. Clinical studies indicate that Resina Draconis can increase levels of MMP-3, VEGF, and TGF-β1 while reducing TIMP-1 to promote wound healing in patients with stress-induced hand injuries [25]. RD capsules can effectively alleviate pain and treat pressure ulcers in elderly patients [26]. Loureirin B (LB), a principal bioactive constituent of Draconis Resina (Dragon’s Blood), significantly enhances extracellular matrix (ECM) synthesis in fibroblasts through activation of the TGF-β/Smad signaling pathway under hyperglycemic conditions. Furthermore, LB administration demonstrates remarkable efficacy in promoting wound healing in diabetic murine models [27]. Although Resina Draconis has proven efficacy in promoting diabetic wound healing, there is limited research on its molecular mechanisms. Therefore, further comprehensive and in-depth studies are essential to clarify the mechanisms by which Resina Draconis promotes diabetic wound healing.

## 2. Materials and Methods

### 2.1. Chemicals and Reagents

Resina Draconis (RD) was procured from Tongren Chinese Herbal Medicine Wholesale Market (Beijing, China) and authenticated by Associate Professor Xiankuan Li (Tianjin University of Traditional Chinese Medicine) as the resin derived from *Dracaena cochinchinensis* (Lour.) S.C.Chen. The chemical constituents of RD were characterized and identified using ultra-performance liquid chromatography coupled with Q-Exactive Orbitrap mass spectrometry (UPLC-Q-Exactive Orbitrap MS) [28]. Notably, the RD material used in this study was from the same batch as that employed in previous compositional analyses conducted in our laboratory.

Mouse mononuclear macrophage leukemia cells (RAW264.7, TCM-C677) were purchased from Suzhou Haixing Biosciences Co., Ltd. (Suzhou, China).

Chemical reagents included: carbomer 940 (Macklin, Shanghai, China), triethanolamine (Macklin, Shanghai, China), ethylparaben (Macklin, Shanghai, China), 1,2-propylene glycol (Aladdin, Shanghai, China), and glycerol (Macklin, Shanghai, China).

Cell culture reagents comprised: RAW264.7 cell-specific medium (Servicebio, Wuhan, China), AGEs-BSA (CY30824, ChemeGen Biotech, Shanghai, China), DMSO (D8371, Solarbio, Beijing, China). The loureirin A (LA), loureirin B (LB), loureirin C (LC), loureirin D (LD), 7,4′-dihydroxyflavone (7,4′-DHF), and resveratrol (RSV) standards (HPLC purity ≥ 98% for all compounds) were purchased from Shanghai Yeasen Biotechnology Co., Ltd. (Shanghai, China).

Materials for animal experiments: Streptozotocin (STZ, Yuanye Biotechnology, Shanghai, China); 60% high-fat diet (Xiaoshu Youtai Co., Ltd., Beijing, China); 2,2,2-tribromoethanol (Aladdin, Shanghai, China); 10% neutral formalin fixative solution (Solarbio, Beijing, China); Masson staining solution (Servicebio, Wuhan, China); recombinant human bFGF (rb-bFGF, Yisheng Bio-pharmaceutical, Shenyang, China); citric acid (Tianjin Huihang Chemical, Tianjin, China); and sodium citrate (Tianjin Fengchuan Chemical Reagent, Tianjin, China).

Experimental kits were obtained as follows: Cell Counting Kit-8 (CCK-8, CA1210, Dalian Meilun Biotechnology, Dalian, China); BCA Protein Assay Kit (PC0020, Solarbio, Beijing, China); TRIzol reagent and reverse transcription kits (T9424, Genstar, Beijing, China).

Antibodies included: anti-RAGE (ab314773, Abcam, Cambridge, MA, USA); anti-AGEs (HY-P81087; MCE, Princeton, NJ, USA); anti-β-tubulin (11224-1-AP; Proteintech, Rosemont, IL, USA); horseradish peroxidase-labeled goat anti-rabbit IgG (GB23303, Servicebio, Wuhan, China); along with anti-iNOS (GB11119, Servicebio, Wuhan, China); anti-CD206 (GB113497, Servicebio, Wuhan, China); and anti-CD68 (GB113109, Servicebio, Wuhan, China).

The instruments used include a microplate reader (BioTek, Winooski, VT, USA), the Amersham ImageQuant™ 800 Protein Imprinting Imaging System (Cytiva, Marlborough, MA, USA), and a high-content screening system (Cytiva, Marlborough, MA, USA).

### 2.2. Preparation of RD Hydrogel

The RD hydrogel was prepared following a previously established method [29]. 1.0 g of Carbomer 940 was slowly dispersed in 20 mL of deionized water, followed by continuous magnetic stirring for 3 h. Approximately 30 mg of ethylparaben was then added as a preservative, after which 0.5 mL of 1,2-propylene glycol and 1 mL of glycerol were incorporated successively. The mixture was neutralized with about 2 mL of triethanolamine. 1 g or 3 g of RD and 0.12 g of LB were separately dissolved in 75% (*v*/*v*) ethanol and then added to the aforementioned hydrogel base. The final formulations were adjusted to 100 g with purified water under vigorous stirring. All hydrogels were stored in sealed containers at 4 °C for subsequent animal experiments.

### 2.3. Research on Network Pharmacology

We identified major active components of RD resin based on our preliminary research and literature review [22,28,30]. These compounds were analyzed using SwissTargetPrediction to identify potential targets. After removing duplicates, we searched GeneCards and OMIM databases using “diabetic wound” and “diabetic ulcer” as keywords to compile disease-related targets. The intersection between compound-predicted targets and disease targets was identified as a potential therapeutic target. The component–target–disease network was visualized using Cytoscape 3.10.0. Protein–protein interaction (PPI) networks were constructed via the STRING database and analyzed in Cytoscape to identify key targets. The key targets were then subjected to GO functional annotation and KEGG pathway enrichment analysis using the DAVID database, with results visualized through the Microbioinformatics platform.

### 2.4. Bioinformatics Analysis of Clinical Data from the GEO Database

For clinical data analysis, gene expression profiles of diabetic wound samples were obtained from the GEO database (accession: GSE29221). Using R software (version 4.5.0) with ggplot2 ( version 3.5.2), factoextra ( version 1.0.7), FactoMineR ( version 2.11), limma (version 3.64.1), and pheatmap (version 1.0.13) packages, we performed PCA, differential gene expression analysis (|logFC| ≥ 2; *p* < 0.05), and cluster analysis. KEGG and GO enrichment analyses followed standard protocols.

### 2.5. Molecular Docking

Molecular docking was conducted between the RAGE protein and the main chemical components of RD that promote diabetic wound healing, as predicted in the “Component–Target–Disease” network. The compounds included LA, LB, LC, LD, RSV, and 7,4′-DHF. The 2D structures (in SDF format) of these ligands were retrieved from PubChem and converted to MOL2 format. The crystal structure of the RAGE receptor (PDB ID: 3O3U) was obtained from the Protein Data Bank (PDB) in PDB format. Molecular docking simulations between the RAGE receptor and the small-molecule ligands were conducted using AutoDockTools 1.5.7, and the results were visualized using PyMOL 3.1.

### 2.6. Cell Culture and Treatment

The RAW264.7 cells underwent cultivation in RAW264.7 cell-specific medium at 37 °C, 5% CO_2_ in a constant temperature incubator. RD extract, along with standard compounds LA, LB, LC, LD, RSV, and 7,4′-DHF, were dissolved in cell culture-grade DMSO to prepare stock solutions with final concentrations of 50 μg/mL, 250 μg/mL, 893.75 μg/mL, 206.25 μg/mL, 1125 μg/mL, 337.5 μg/mL, and 662.5 μg/mL, respectively. The dosage is derived from conversion based on content determination [28]. AGEs-BSA powder was dissolved in PBS solution to prepare the AGEs-BSA stock solution.

The impact of AGEs-BSA and RD on the survival of RAW264.7 cells was assessed using the CCK-8 assay. RAW264.7 cells were seeded in 96-well plates at a density of 2 × 10^4^ cells/well and incubated at 37 °C for 24 h. Subsequently, the cells underwent treatment with AGEs-BSA in concentrations of 0, 2.5, 50, 100, 200, and 500 μg/mL for 24 h to assess cytotoxicity. Similarly, the cells were incubated with RD solution in 0, 0.781, 1.563, 3.123, 6.25, 12.5, 25, and 50 μg/mL. After 24 h, 10 μL CCK-8 solution was added to each well, and incubated in a constant temperature incubator at 37 °C for 1 h in the dark. The absorbance at 450 nm was measured using a microplate reader.

After dose optimization, the experiment was designed with: (1) untreated control, (2) AGEs-BSA model group (200 μg/mL), (3) RD treatment groups (6.25, 3.13, and 1.56 μg/mL), or LA, LB, LC, LD, 7,4′-DHF, and RSV treatment groups. RAW264.7 cells were plated in 96-well plates at a 1 × 10^6^ cells/mL density and then treated with either AGEs-BSA alone or in combination with the specified concentrations of RD or reference compounds for 24 h before further analysis.

### 2.7. Western Blot (WB) Analysis

RAW264.7 cells were lysed with RIPA buffer containing phosphatase inhibitors. Cell lysates were centrifuged at 12,000 rpm for 10 min at 4 °C, and the supernatants were collected. The protein concentrations in the supernatants were measured using a BCA Protein Assay Kit. Approximately 20 μg of the protein samples were separated on 10% SDS-PAGE and transferred to polyvinylidene difluoride (PVDF) membranes. The membranes were blocked with 5% (*w*/*v*) skim milk in TBST (1× Tris Buffered saline, 0.1% TWEEN 20) for 2 h at room temperature. The blocked membranes were incubated with the following primary antibodies: RAGE, β-Tubulin, at 4 °C overnight. Then, the membranes were washed with TBST and probed with horseradish peroxidase-labeled Goat anti-rabbit IgG, which was used as the secondary antibody, for 1 h at room temperature. Blot bands were visualized using an ECL detection Kit. The Amersham ImageQuant TM 800 Protein Imprinting Imaging System was employed to observe protein bands. Using ImageJ software (version 1.53t), the grayscale measurements of the bands were quantified.

### 2.8. RNA Extraction and Quantitative RT-PCR Analysis

Total RNA was extracted from cells using the TRIzol reagent kit according to the manufacturer’s protocol. Subsequently, 1 μg of RNA was reverse transcribed into cDNA using a reverse transcription kit. The synthesized cDNA was then subjected to quantitative real-time PCR (qPCR) amplification using SYBR Green chemistry. Primer sequences are listed in Table 1, and β-actin was used as the reference gene. The relative mRNA expression levels of target genes were calculated using the 2^−ΔΔ^^Ct^ method.

### 2.9. Measurement of ROS

RAW264.7 cells were seeded in 96-well plates at 1 × 10^4^ cells/well and incubated at 37 °C for 24 h for adhesion. Cells were divided into control, model, high/medium/low-dose RD extract, and LB treatment groups (*n* = 3). After replacing with drug-containing medium, cells were cultured for another 24 h. Supernatants were discarded, washed twice with PBS, and incubated with 10 μM H2DCFDA in the dark (37 °C, 30 min). Post-washing, 200 μL D’Hanks buffer was added per well. Images were captured using a high-content screening system, and fluorescence intensity was quantified with ImageJ.

### 2.10. Construction of the Diabetic Wound Mouse Model and Group Administration

A total of 130 male C57BL/6J mice aged 6–8 weeks were supplied by Huafukang Biotechnology Co., Ltd. (Beijing, China). All mice were housed in a specific pathogen-free (SPF) environment under a 12 h light/dark cycle, with free access to standard chow and water during a one-week acclimatization period. The mice were randomly divided into two groups: the diabetes group (*n* = 110) was fed with a high-fat-high-sucrose diet (HFD) for 4 weeks, followed by intraperitoneal injection of streptozotocin (STZ, 55 mg/kg, dissolved in 0.1 mol/L sodium citrate buffer, pH 4.2–4.5) for 7 consecutive days to induce a type 2 diabetes mellitus (T2DM) model, while the control group (*n* = 20) received standard chow diet and was injected with an equal volume of citrate buffer. One week after the last injection, mice with fasting blood glucose levels > 16.7 mmol/L were considered to have successfully developed STZ-induced diabetes. Ninety mice that met the diabetic criteria were used for subsequent experiments. Figure S3 shows the blood glucose levels of mice in both the control and model groups at weeks 1, 2, and 3 after STZ injection.

The mice were anesthetized with 2% 2,2,2-tribromoethanol, and their back hair on the dorsal skin (about 300 mm^2^ area) was shaved. After the hairless area was disinfected with iodine tincture, a full-thickness excision wound was created using a punch biopsy device with a sterile diameter of 8 mm. The mice were divided into six groups (*n* = 18) as follows: (1) control wound-vehicle group (control, blank hydrogel treatment group), (2) diabetic wound-vehicle group (model, blank hydrogel treatment group), (3) diabetic wound-3% RD hydrogel (RD-H Gel, 3% RD hydrogel treatment group), (4) diabetic wound-1% RD hydrogel (RD-L Gel, 1% RD hydrogel treatment group), (5) diabetic wound-loureirin B (LB Gel, 0.12% loureirin B hydrogel treatment group), (6) diabetic wound-rbFGF (rb-bFGF Gel, rb-bFGF hydrogel treatment group). The dosage of LB was determined based on the content analysis from our previous study, calculated by converting the amount used in the RD-H Gel group [28]. Rb-bFGF is a commonly used wound healing growth factor, so it was selected as a positive control to verify the therapeutic effect of RD. On days 0, 3, 5, 10, and 14 after wound induction, the area of the wound surface was photographed and measured using ImageJ software. On days 3, 5, and 14 after the operation, six mice in each group were euthanized, and the skin tissue around the wound was collected for histological and biochemical analysis.

### 2.11. Hematoxylin-Eosin Staining (H&E)

The tissues were fixed in 10% neutral formalin fixative solution for 24 h, followed by standard paraffin embedding and sectioning. After sequential dewaxing and rehydration through a graded ethanol series, sections were subjected to H&E staining (hematoxylin: 3–5 min; eosin: 15 s). Paraffin sections were sequentially dehydrated through an ethanol gradient, cleared in xylene, and mounted with neutral balsam for microscopic examination.

### 2.12. Masson’s Trichrome Staining

After dewaxing and rehydration through a graded ethanol series, paraffin sections were stained overnight in Masson A solution. Following brief washing, sections were treated with Masson B/C mixture (1:1, 1 min), differentiated, and sequentially stained with Masson D (6 min) and E (1 min) solutions. After 230 s in Masson F solution, sections were differentiated in 1% acetic acid, dehydrated in absolute ethanol, cleared in xylene, and mounted with neutral resin.

### 2.13. Immunohistochemistry

After dewaxing through graded xylene and ethanol series, antigen retrieval was conducted using optimal pH buffer (citrate/EDTA) with temperature control. Endogenous peroxidase activity was blocked with 3% H_2_O_2_ (25 min, RT), followed by protein blocking with 3% BSA (30 min). Primary antibody RAGE and AGEs incubation was carried out overnight at 4 °C in a humidified chamber, followed by appropriate HRP-conjugated secondary antibody (50 min, RT). Signal development was achieved using DAB substrate with real-time microscopic monitoring, and nuclei were counterstained with hematoxylin (3 min). Finally, sections were dehydrated through graded ethanol, cleared in xylene, and mounted with resin for microscopic analysis. All washing steps were performed using PBS (pH 7.4) with gentle agitation.

### 2.14. Immunofluorescence

Paraffin sections were dewaxed in eco-friendly solutions I–III (10 min each), dehydrated in absolute ethanol I–III (5 min each), and rinsed. Antigen retrieval was performed per pre-set conditions, followed by cooling and PBS washing. Tissue areas were marked with a hydrophobic pen, and endogenous peroxidase was blocked using 3% H_2_O_2_ for 25 min in the dark, then washed with PBS. Sections were blocked with 3% BSA (or 10% rabbit serum for goat primary antibodies) for 30 min. Primary antibodies iNOS and CD206 were separately applied overnight at 4 °C, followed by HRP-conjugated secondary antibody for 50 min at RT, TSA incubation for 10 min in the dark, and TBST washes. After microwave treatment with the retrieval buffer, the primary antibody 2 CD68 was applied overnight at 4 °C. For fluorescence detection, either HRP-TSA or directly conjugated antibodies were used. Nuclei were counter-stained with DAPI for 10 min in the dark, and autofluorescence was quenched if needed. Finally, antifade-mounted slides were imaged with DAPI/488/Cy3/Cy5 filters.

### 2.15. Statistical Analysis

Statistical analysis was explored using SPSS 26.0 software (SPSS, Chicago, IL, USA). Data were analyzed using one-way analysis of variance (ANOVA). The significance level was determined as *p* ≤ 0.05. Results are expressed as mean ± standard deviation (SD).

## 3. Results

### 3.1. Analysis of Network Pharmacology

**Figure 1:**
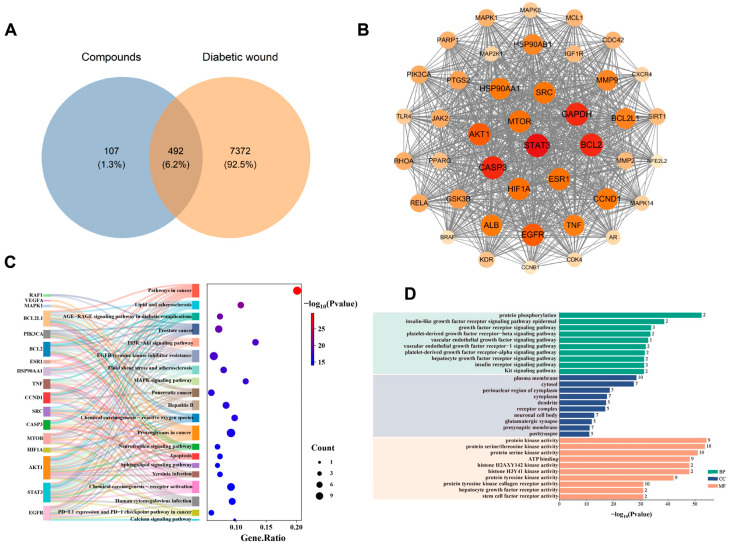
Mechanism prediction of RD in promoting diabetic wound healing: (**A**) Venn diagram of RD components and DW targets. (**B**) Protein–protein interaction network of 42 effective targets. (**C**) Bubble chart of KEGG enrichment analysis for target genes. (**D**) Bar graph of GO enrichment analysis for target genes.

We have identified a total of 42 components of RD for network pharmacology analysis (Table S1). Through the SwissTargetPrediction database, we identified 599 potential targets associated with 42 active compounds derived from Resina Draconis (RD). Concurrently, a comprehensive search of the GeneCards and OMIM databases using “diabetic wound” and “diabetic ulcer” as keywords yielded 7864 disease-related targets after deduplication. The intersection analysis revealed 492 common targets between the compound-related and disease-related targets (Figure 1A), which were identified as potential therapeutic targets for RD in promoting diabetic wound healing. The comprehensive “Compound–Target–Disease” network visually illustrates the intricate relationships between the bioactive components and their corresponding therapeutic targets (Figure S1). High-degree compounds include resveratrol (RSV), loureirin B (LB), loureirin A (LA), loureirin C (LC), loureirin D (LD), 7,4′-dihydroxyflavone (7,4′-DHF), apigenin, pterostilbene, p-hydroxybenzoic acid, and isoliquiritigenin. These components are considered the main chemical constituents in RD that promote diabetic wound healing. We screened 42 core targets (Table S2) from the initial 492 overlapping targets using three topological parameters: degree centrality (92), betweenness centrality (0.00913), and closeness centrality (0.53509). The constructed protein–protein interaction (PPI) network (Figure 1B) consisted of 42 nodes and 794 edges, with key hub targets including STAT3, BCL2, CASP3, AKT1, and EGFR. These central targets are known to play pivotal roles in wound healing processes: STAT3 modulates inflammatory responses and macrophage polarization [31]; BCL2 regulates apoptosis in various wound cells [32]; CASP3 is involved in both apoptosis and keratinocyte migration [33]; AKT1 promotes cell survival and angiogenesis through the PI3K-Akt pathway [34]; and EGFR signaling is crucial for epithelial proliferation and regeneration [35].

The KEGG enrichment results (Figure 1C) showed that the core targets were mainly involved in pathways related to lipid and atherosclerosis, the AGE-RAGE signaling pathway in diabetic complications, the PI3K/Akt signaling pathway, etc. Notably, the significant enrichment of the AGE-RAGE and PI3K-Akt pathways suggests that RD may alleviate diabetic wounds by mitigating hyperglycemia-induced inflammatory damage and promoting cellular survival and angiogenesis. The GO enrichment results (Figure 1D) indicated that the core targets were mainly distributed in the plasma membrane, cytosol, perinuclear region of cytoplasm, and other compartments, and were involved in signaling pathways such as protein phosphorylation, insulin-like growth factor receptor signaling pathway, epidermal growth factor receptor signaling pathway, platelet-derived growth factor receptor-beta signaling pathway, etc. These findings imply that RD’s therapeutic effects may be mediated through regulating membrane receptor-mediated signaling, intracellular phosphorylation events, and growth factor responses that are essential for coordinated wound repair.

### 3.2. Clinical Sample Analysis

**Figure 2:**
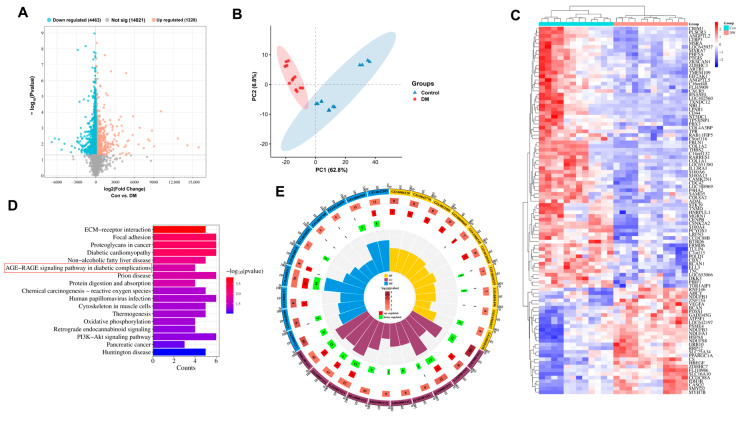
Bioinformatics analysis of clinical samples from the GSE29221 dataset: (**A**) Volcano plot displays differentially expressed genes (DEGs) with statistical significance. (**B**) Principal component analysis (PCA) demonstrates sample clustering based on gene expression profiles. (**C**) Hierarchical clustering heatmap illustrates expression patterns of key DEGs across samples. (**D**) KEGG pathway analysis reveals significantly enriched biological pathways among major DEGs. (**E**) Gene Ontology (GO) enrichment analysis identifies statistically significant functional terms in biological processes, molecular functions, and cellular components.

This study included 24 samples from the GSE29221 dataset for analysis. Through systematic screening, a total of 401 upregulated genes and 107 downregulated genes were identified (Figure 2A). The results of principal component analysis (PCA) showed significant separation in gene expression profiles between the control group and the diabetic wound group, indicating obvious biological differences between the two groups (Figure 2B). Further hierarchical clustering analysis of the GSE29221 dataset focused on the top 100 differentially expressed genes, including key genes such as *S100A6*, *CSNK2A2*, *TNC*, *HBEGF*, *PCYOX1*, *FRMD6*, and *CDC42* (Figure 2C). These genes may play important regulatory roles in the pathological process of diabetic wounds: *S100A6* is involved in calcium-binding and inflammatory responses [36]; *CSNK2A2* regulates multiple signaling pathways through phosphorylation [37]; *TNC* contributes to extracellular matrix organization and cell adhesion [38]; *HBEGF* stimulates keratinocyte and fibroblast proliferation [39]; *PCYOX1* is associated with oxidative stress [40]; *FRMD6* participates in cell polarity establishment [41]; and *CDC42* regulates cell motility and angiogenesis [42]. These genes may play important regulatory roles in the pathological process of diabetic wounds. KEGG pathway analysis (Figure 2D) revealed that genes related to diabetic wounds were significantly enriched in ECM-receptor interaction, focal adhesion, proteoglycans in cancer, diabetic cardiomyopathy, and the AGE-RAGE signaling pathway in diabetic complications. The enrichment of ECM-receptor interaction and focal adhesion pathways indicates profound disruption in cell–matrix communication, while the emergence of AGE-RAGE signaling underscores the importance of metabolic memory in diabetic wound pathogenesis. GO enrichment analysis (Figure 2E) indicated that differentially expressed proteins were mainly distributed in subcellular structures such as mitochondria, cell membrane, and extracellular matrix, participating in multiple important biological pathways, including mitochondrial electron transport from NADH to ubiquinone, macrophage differentiation, and extracellular matrix organization. These results suggest that diabetic wounds exhibit impairments in energy metabolism, immune cell differentiation, and structural organization at the subcellular level.

Based on the results of network pharmacology prediction and bioinformatics analysis of clinical samples, which both pinpointed the AGE-RAGE signaling pathway as a critical nexus, this study took the AGE-RAGE signaling pathway as the key research object and will further explore the molecular mechanism and potential intervention targets of diabetic wounds around this pathway in the follow-up research.

### 3.3. The Main Components of RD Can Tightly Bind to the Extracellular Domain of RAGE

**Figure 3:**
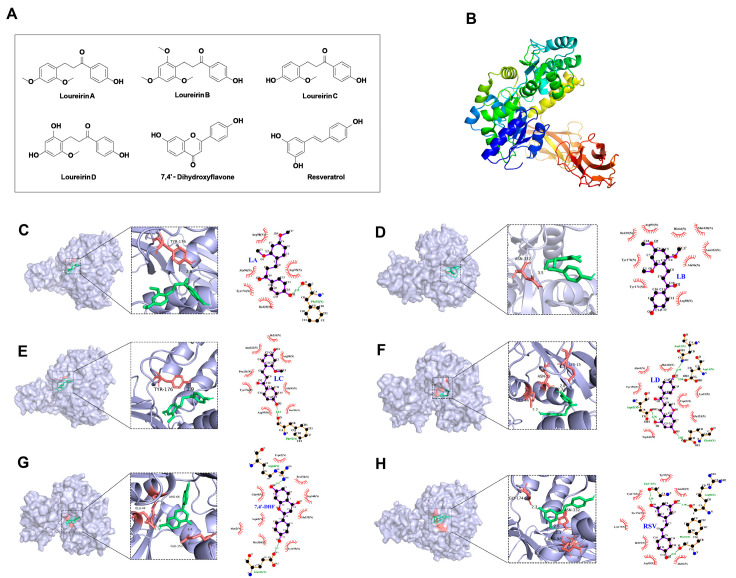
Molecular docking analysis of drug–target binding interactions: (**A**) Chemical structure of the main active components in RD. (**B**) Schematic diagram of the 3D structure of RAGE protein (3O3U) selected from the PDB database. (**C**–**H**) Binding con-formations of RAGE protein with active ingredient ligands: (**C**) LA, (**D**) LB, (**E**) LC, (**F**) LD, (**G**) 7,4′-dihydroxyflavone (7,4′-DHF), and (**H**) resveratrol (RSV). The most stable conformation with the lowest binding energy was selected as the optimal binding mode for each compound.

Based on our quantitative analysis results [28], RSV, LB, LA, LC, LD, and 7,4′-DHF were the components with the highest content. Additionally, the Compound–Core–Target–Disease network analysis (Figure S1) indicated that these compounds were key. Therefore, RSV, LB, LA, LC, LD, and 7,4′-DHF were selected for molecular docking studies (Figure 3A). The binding energies of LA, LB, LC, LD, 7,4′-DHF, and RSV to RAGE (Figure 3B) were −5.79 kcal/mol, −6.20 kcal/mol, −6.55 kcal/mol, −7.78 kcal/mol, −8.99 kcal/mol, and −6.80 kcal/mol, respectively. Molecular docking analysis (Figure 3C–H) demonstrated strong interactions between bioactive compounds and RAGE’s extracellular domain, with binding energies below −5 kcal/mol. The flavonoid 7,4′-DHF showed particularly high affinity (−8.99 kcal/mol). Structural studies revealed that RSV, LB, LA, LC, LD, and 7,4′-DHF fit precisely into RAGE’s binding pocket, exhibiting optimal shape complementarity. Key residues (TYR-176, ASN-332, and GLU-44) formed multiple hydrogen bonds with ligand oxygen atoms, significantly enhancing complex stability.

### 3.4. RD and Its Active Components Attenuate AGEs-Induced RAGE Upregulation

We first determined the optimal concentrations of AGEs-BSA and RD. No significant cytotoxicity was observed when the concentration of AGEs-BSA was in the range of 25–500 μg/mL. RD at concentrations of 50, 25, and 12.5 μg/mL significantly inhibited cell proliferation, while concentrations ≤ 6.25 μg/mL showed no inhibitory effect. Therefore, we selected 200 μg/mL AGEs-BSA and three concentrations of RD (6.25, 3.13, and 1.56 μg/mL) for subsequent experiments.

**Figure 4:**
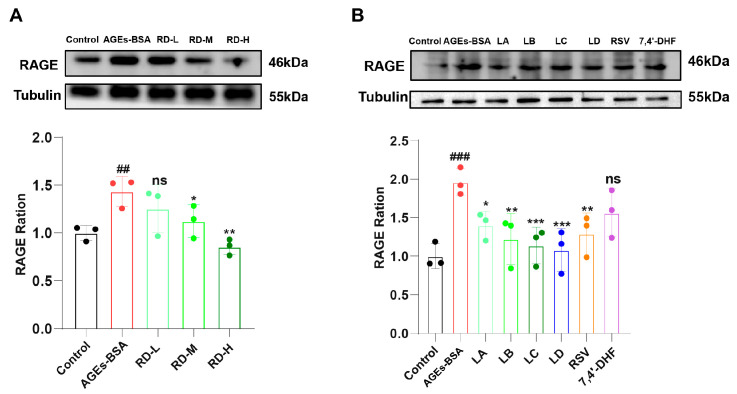
Effects of AGEs-BSA, RD extract, and RD compounds on RAGE protein expression. (**A**,**B**) Relative RAGE protein levels in RAW264.7 cells, normalized to β-Tubulin as a loading control. (*n* = 3). Data were presented as mean ± standard deviation. ^##^ *p* < 0.01, ^###^ *p* < 0.001, vs. Control group; * *p* < 0.05, ** *p* < 0.01, *** *p* < 0.001, vs. model group; ns indicates no significant difference.

Western blot analysis revealed that 200 μg/mL AGEs-BSA significantly increased RAGE protein expression (*p* < 0.01), which was effectively reversed by RD treatment in a dose-dependent manner (1.56–6.25 μg/mL) (Figure 4A). Then, the effects of the major RD constituents (LA, LB, LC, LD, RSV, and 7,4′-DHF) on RAGE expression were evaluated at doses of 87.31, 282.52, 75.74, 390.23, and 290.26 nM, respectively. These doses were determined based on the content of these components in RD [28]. Compared with the AGEs-BSA group, the LA group (*p* < 0.05) could significantly inhibit RAGE expression; the LB group and RSV group (*p* < 0.01) could effectively down-regulate RAGE levels; the LC group and LD group (*p* < 0.001) showed better inhibitory effects; whereas no significant regulatory effect on RAGE expression was observed in the 7,4′-DHF group (Figure 4B). Additionally, PCR results confirmed that RD (6.25, 3.13, and 1.56 μg/mL) and LB (the most abundant component) significantly downregulated RAGE mRNA expression (Figure S2).

### 3.5. RD and LB Reduce Inflammatory Responses and ROS Generation

**Figure 5:**
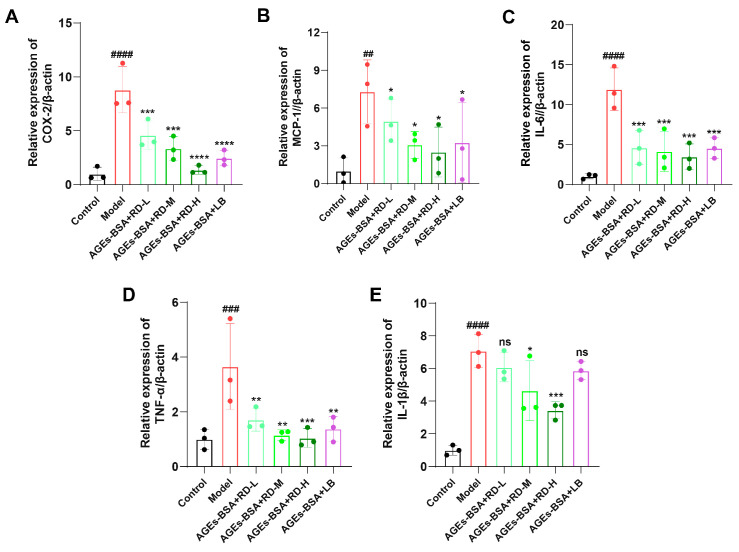
Quantification of inflammatory cytokine mRNA expression levels by qPCR. (**A**–**E**) Effects of RD and LB on mRNA levels of COX-2, MCP-1, IL-6, TNF-α, and IL-1β in AGEs-BSA-treated RAW264.7 cells (*n* = 3). Data were presented as mean ± standard deviation. ^##^ *p* < 0.01, ^###^ *p* < 0.001, ^####^ *p* < 0.0001, vs. Control group; * *p* < 0.05, ** *p* < 0.01, *** *p* < 0.001, **** *p* < 0.0001, vs. model group; ns indicates no significant difference.

The combination of AGEs and RAGE activated the inflammatory response of cells, resulting in increased gene expression levels of inflammatory factors. We found that all major components of RD (LA, LB, LC, LD, RSV, and 7,4′-DHF) can downregulate the expression of RAGE protein. Therefore, we selected RD and LB (the most abundant component of RD) for studies on inflammation and oxidative stress. The dose of LB was 835.8 nM, which was equal to the total combined amount of LA, LB, LC, and LD. Here, we determined the mRNA expression levels of IL-6, IL-1β, TNF-α, COX-2, and MCP-1 in RAW264.7 cells (Figure 5A–E). AGEs-BSA (200 μg/mL) significantly increased mRNA levels of these inflammatory factors in RAW264.7 cells. RD treatment dose-dependently reduced these inflammatory factors. Similarly, LB also significantly reduced the expression levels of the five inflammatory factors, but the inhibitory effect was not as good as that of RD administration.

**Figure 6:**
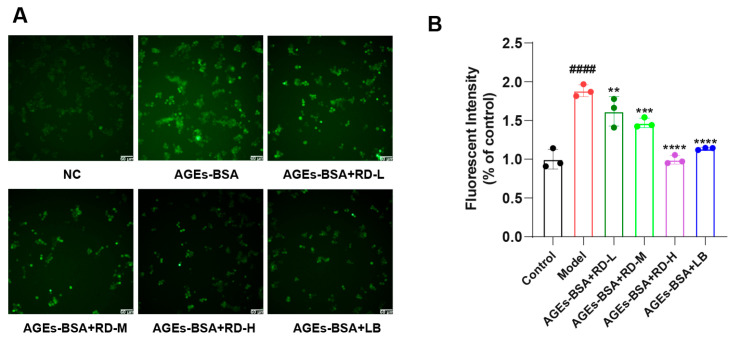
Results of ROS generation assays: (**A**) Representative fluorescence images of ROS generation. (**B**) Measurement of ROS generation after intervention with different doses of RD and LB (*n* = 3). Data were presented as mean ± standard deviation. ^####^ *p* < 0.0001, vs. Control group; ** *p* < 0.01, *** *p* < 0.001, **** *p* < 0.0001, vs. model group; ns indicates no significant difference.

The cell images were collected by a high-content screening instrument, and the fluorescence intensity of each group was calculated with ImageJ. The results (Figure 6A) showed that 200 μg/mL AGEs-BSA significantly stimulated ROS production in RAW264.7 cells, while RD dose-dependently inhibited this effect. LB (835.8 nM) significantly reduced ROS production, but its inhibitory effect was weaker than that of RD (Figure 6B).

### 3.6. RD and LB Promote Wound Healing in Diabetic Mice During the Inflammatory Phase

**Figure 7:**
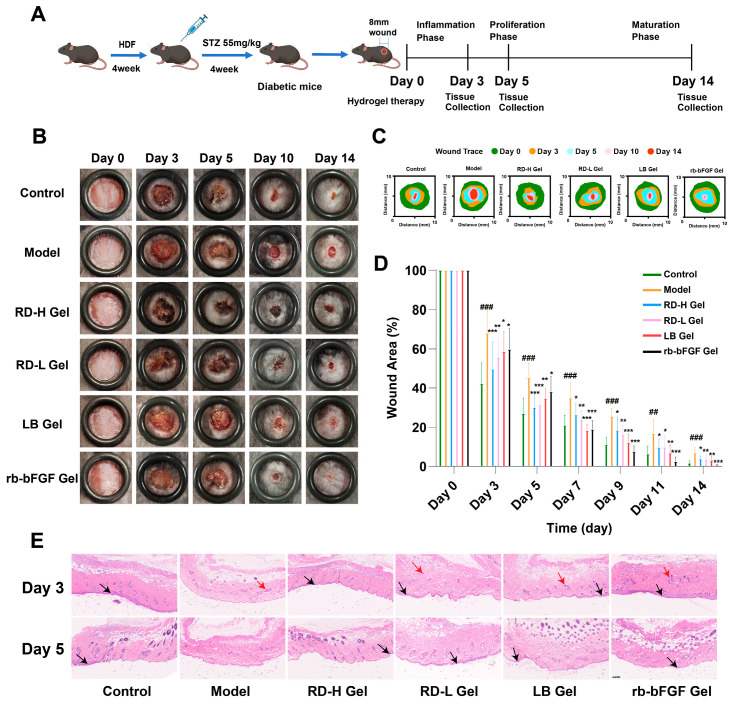
Evaluation of RD and LB in promoting diabetic wound healing in vivo: (**A**) Schematic diagram of the diabetic wound healing experimental procedure. (**B**,**C**) Representative images and schematic diagrams of wound healing at days 0, 3, 5, 10, and 14 post-surgery for the Control group, model group, RD-H Gel group, RD-L Gel group, LB Gel group, and rb-bFGF Gel group under the same treatment conditions. (**D**) Wound area analysis using ImageJ software, normalized to the wound area on day 0 (*n* ≥ 6). (**E**) Representative HE staining results of skin wound sections on days 3 and 5 post-surgery (*n* = 3, scale bar: 0.2 mm). Black arrows indicate neo-epithelium (re-epithelialization). Red arrows indicate areas of inflammatory cell infiltration (predominantly neutrophils). Data were presented as mean ± standard deviation. ^##^ *p* < 0.01, ^###^ *p* < 0.001, vs. Control group; * *p* < 0.05, ** *p* < 0.01, *** *p* < 0.001, vs. model group.

To further verify whether RD and its main component, LB, have medicinal effects and to investigate whether the medicinal effects are related to the AGE-RAGE pathway, we performed animal experiments. The flowchart is shown in Figure 7A. As shown in Figure 7B–D, on days 3 and 5, the wound healing in the control group was the most obvious, while the mice in the model group had higher blood glucose levels and exhibited the typical characteristic of delayed healing of diabetic wounds (*p* < 0.001). The RD hydrogel treatment groups significantly promoted wound healing, with the RD-H Gel group exhibiting a more pronounced effect. Additionally, the LB Gel group also demonstrated notable efficacy on day 3 (*p* < 0.05) and day 5 (*p* < 0.01). In contrast, the positive control group showed relatively weaker effects (*p* < 0.05). HE staining results (Figure 7E) on days 3 and 5 revealed a markedly impaired healing process in the model group, characterized by thinner neo-epithelium, excessive inflammatory cell infiltration, and tissue disorganization, compared to the control group. In contrast, the RD-H and LB treatment groups exhibited accelerated re-epithelialization and reduced inflammation, which corroborated the macroscopic wound closure data.

From day 7 onward, the control group maintained the highest wound closure rate and fastest healing speed, followed by the positive control and LB groups, while the RD hydrogel groups exhibited slower progression. By day 14, the control group achieved the fastest healing, with the positive control group showing significantly improved recovery versus the model group (*p* < 0.001). Both the RD-L group and the LB group also demonstrated marked therapeutic effects (*p* < 0.01).

**Figure 8:**
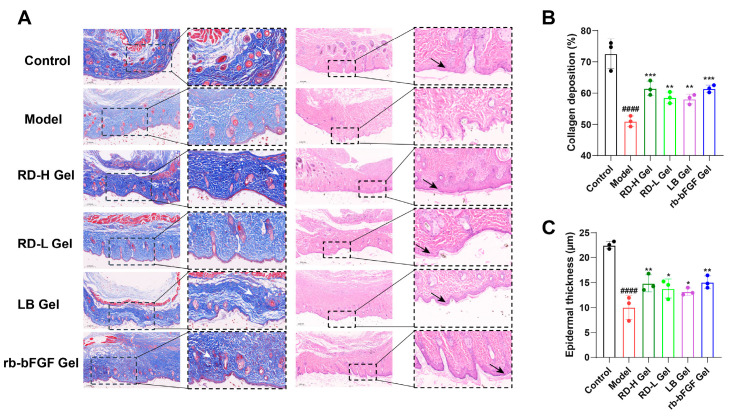
Evaluation of RD and LB in promoting diabetic wound healing in vivo: (**A**) Representative HE and Masson staining results of skin wound sections on day 14 post-surgery. Black arrows indicate the fully regenerated epidermis (re-epithelialization). White arrows indicate dense and well-organized blue collagen fibers. (**B**,**C**) Quantitative analysis of neoepithelial thickness and collagen deposition (*n* = 3). Data were presented as mean ± standard deviation. ^####^ *p* < 0.0001, vs. Control group; * *p* < 0.05, ** *p* < 0.01, *** *p* < 0.001, vs. model group.

Histopathological analysis of mouse skin tissues at day 14 (Figure 8A) using H&E and Masson’s trichrome staining revealed significant morphological differences among groups. The control group maintained an intact epidermal structure with well-defined stratification and uniformly distributed collagen fibers exhibiting normal architecture. In contrast, the model group demonstrated characteristic pathological features, including epidermal thinning, tissue disorganization, and reduced/disordered collagen deposition. Therapeutic intervention with various gel formulations showed significant restorative effects, manifested by improved epidermal morphology, normalized cellular arrangement, increased collagen content, and more organized fiber alignment.

Quantitative analysis demonstrated that RD-H Gel treatment significantly enhanced collagen deposition (Figure 8B) and restored epidermal thickness (Figure 8C), with efficacy comparable to the positive control (rb-bFGF Gel), suggesting its potent regenerative capacity for skin tissue repair.

In summary, throughout the observation period, the control group displayed the most rapid and complete wound healing, whereas the model group healed the slowest. During the early phase, the RD treatment groups outperformed both the model and positive control groups. In later stages, all treatment groups exhibited significant efficacy, with the positive control demonstrating the strongest effects. As shown in Figure 7D, RD hydrogel demonstrated pronounced early-phase anti-inflammatory effects (days 3–5), contributing to significantly accelerated initial wound closure, while rb-bFGF primarily facilitated later proliferative processes, enhancing tissue regeneration during the final stages of healing.

### 3.7. RD and Its Bioactive Components Significantly Downregulate AGE and RAGE Expression During the Inflammatory Phase

**Figure 9:**
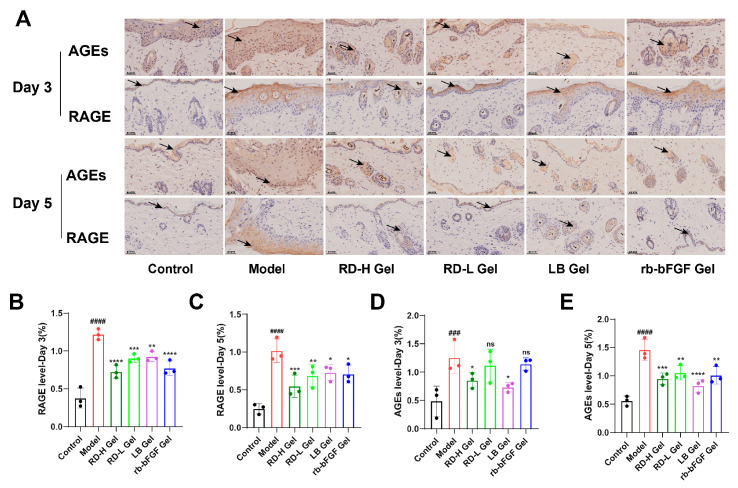
Evaluation of the regulatory effects of RD and LB on the AGE-RAGE axis in vivo. (**A**) Immunohistochemical staining showing AGEs and RAGE expression (brownish-yellow indicates positive staining) in tissues from each group at Day 3/Day 5 (Scale bar: 0.05 mm). Black arrows indicate cells with intense brownish-yellow positive staining for AGEs/RAGE, primarily localized in the cytoplasm and cell membrane. (**B**–**E**) Semi-quantification of immunohistochemical staining by positive area percentage (*n* = 3). ^###^ *p* < 0.001, ^####^ *p* < 0.0001 vs. Control group; * *p* < 0.05, ** *p* < 0.01, *** *p* < 0.001, **** *p* < 0.0001 vs. model group; ns indicates no significant difference.

Immunohistochemical analysis (Figure 9A) revealed significantly enhanced deposition of AGEs and RAGE in the model group relative to the control group at both day 3 and day 5. Semi-quantitative evaluation (Figure 9B–E) demonstrated markedly elevated expression levels of AGEs (*p* < 0.001 at day 3; *p* < 0.0001 at day 5) and RAGE (*p* < 0.0001 at day 3; *p* < 0.0001 at day 5) in the model group. Therapeutic interventions produced distinct response patterns, with RD-H Gel showing the most robust and sustained suppression of both AGEs (*p* < 0.05 at day 3; *p* < 0.001 at day 5) and RAGE (*p* < 0.001 at day 3; *p* < 0.0001 at day 5), while RD-L Gel and LB Gel exhibited more time-dependent effects, achieving significant RAGE reduction by day 5 (*p* < 0.01).

### 3.8. RD and Its Active Components Induce M2 Macrophage Polarization While Suppressing AGEs-Induced M1 Macrophage Activation

**Figure 10:**
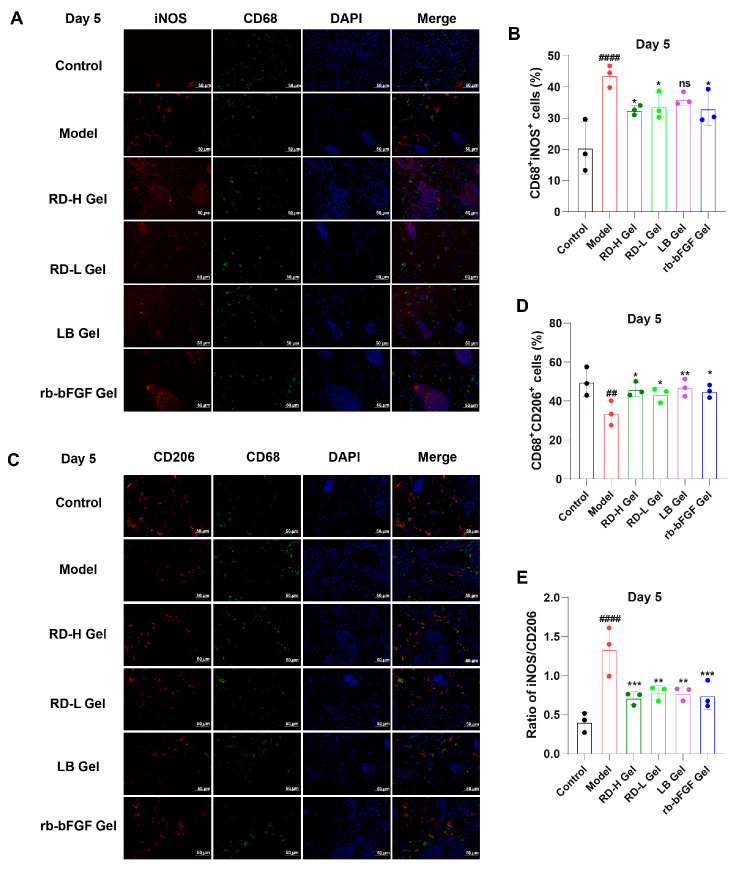
Evaluation of the regulatory effects of RD and LB on macrophage polarization in vivo: (**A**) Immunofluorescence staining of iNOS^+^ (M1, red), CD68^+^ macrophages (green), and DAPI (blue) at day 5 (Scale bar: 50 μm). (**B**) Semi-quantitative analysis of M1 macrophages (CD68^+^iNOS^+^ cells). (**C**) Immunofluorescence staining of CD206^+^ (M2, red), CD68^+^ macrophages (green), and DAPI (blue) at day 5 (Scale bar: 50 μm). (**D**) Semi-quantitative analysis of M2 macrophages (CD68^+^CD206^+^ cells). (**E**) Semi-quantitative analysis of the M1/M2 macrophage ratio. (For B–E: *n* = 3; data are presented as mean ± SD). ^##^ *p* < 0.01, ^####^ *p* < 0.0001 vs. Control group; * *p* < 0.05, ** *p* < 0.01, *** *p* < 0.001 vs. model group; ns indicates no significant difference.

Dual-labeling immunofluorescence revealed dynamic changes in macrophage phenotypes during wound healing (Figure 10). At day 3, the model group exhibited significantly increased infiltration of pro-inflammatory M1 macrophages (CD68^+^iNOS^+^) compared to controls, while treatment groups (RD-H Gel, RD-L Gel, etc.) showed preliminary reduction in M1 infiltration. Concurrently, the model group demonstrated severely impaired polarization of anti-inflammatory M2 macrophages (CD68^+^CD206^+^), whereas treatment groups displayed slightly stronger CD206^+^ signals, suggesting early attempts at phenotypic switching toward reparative states (Figure S4).

By day 5, the model group maintained elevated M1 levels (CD68^+^iNOS^+^), evidenced by persistent red fluorescence and abundant double-positive cells, confirming sustained inflammation. In stark contrast, treatment groups exhibited marked, formulation-dependent reductions in M1 infiltration. The RD-H Gel group showed the most pronounced decline in CD68^+^iNOS^+^ cells, with significantly lower fluorescence intensity and cell counts than the model group (*p* < 0.05), demonstrating potent mitigation of M1-dominant inflammation (Figure 10A,B). Regarding M2 polarization (CD68^+^CD206^+^), the model group remained deficient with sparse CD206 signals, while RD-H Gel and RD-L Gel groups displayed substantial enhancement, featuring increased double-positive cells and intensified red fluorescence, reflecting effective M2 conversion (Figure 10C,D).

Quantitative analysis (Figure 10E) highlighted that the model group maintained a high M1/M2 ratio throughout, whereas RD-H Gel treatment restored near-normal M1/M2 balance by day 5 (*p* < 0.001). This sharp contrast between the model group (with persistent M1 bias) and treatment groups (particularly RD-H Gel, showing accelerated M1 resolution and M2 promotion) underscores the latter’s efficacy by modulating macrophage polarization to resolve inflammation promptly.

## 4. Discussion

Impaired healing of diabetic wounds represents a significant clinical challenge characterized by complex and interconnected pathological mechanisms, including persistent inflammatory responses, excessive oxidative stress, dysfunctional angiogenesis, and abnormal cellular metabolism [43]. This dynamic process encompasses four distinct phases. Hemostasis Phase: When the body is injured, the hemostasis phase begins immediately. Damaged blood vessels trigger platelet aggregation, forming a plug to stop bleeding. Vasoconstriction reduces blood flow, while activated clotting factors strengthen the clot with fibrin. This phase starts within minutes and lasts hours. Inflammatory Phase: Following hemostasis, inflammation begins. Immune cells migrate to the wound to clear bacteria and debris, causing redness, swelling, and pain. This phase typically lasts 3–5 days, depending on wound severity. Proliferative Phase: Next, fibroblasts proliferate, producing collagen and extracellular matrix to fill the wound. New capillaries form to supply nutrients, promoting wound contraction. This stage lasts weeks to months. Remodeling Phase: Finally, collagen is reorganized, balancing synthesis and degradation to strengthen the scar. The tissue gradually regains near-normal structure and function, a process that may take months to years [44,45,46].

The pathological prolongation of the inflammatory phase is a key factor contributing to impaired wound healing in diabetes [47], primarily due to persistent M1 macrophage polarization, elevated pro-inflammatory cytokines (TNF-α, IL-6, IL-1β), ROS overproduction, and sustained AGE-RAGE signaling under chronic hyperglycemic conditions [48,49]. Critically, this chronic inflammatory state creates a hostile microenvironment that directly suppresses subsequent proliferative processes. The pro-inflammatory cytokines and oxidative stress inhibit fibroblast function and collagen synthesis, impair the proliferation and migration of keratinocytes necessary for re-epithelialization, and disrupt the balance of angiogenic factors, leading to inadequate blood vessel formation [50]. RAGE, an immunoglobulin superfamily pattern recognition receptor, critically mediates diabetic wound healing pathology by acting as a central hub that amplifies and perpetuates inflammation and oxidative stress. Encoded by a gene on human chromosome 6, RAGE comprises 404 amino acids with distinct structural domains: an intracellular domain, transmembrane region, and three immunoglobulin-like extracellular domains [51]. While initially identified as the receptor for AGEs, subsequent research has revealed its capacity to bind diverse ligands, including HMGB1 and S100/Aβ proteins, establishing its role as a molecular pattern recognition receptor in inflammatory and immune regulation [10,52]. Its activation triggers a cascade of downstream events, primarily through the NF-κB pathway, which not only fuels inflammation but also contributes to the dysfunction of multiple cell types critical for healing, including endothelial cells (impairing angiogenesis) and fibroblasts (impairing collagen deposition) [53].

The current study demonstrates that excessive activation of the AGE-RAGE signaling pathway represents one of the core mechanisms underlying diabetic wound healing impairment. Through integrated network pharmacology and GEO database analysis, we identified that RD’s potential therapeutic targets are predominantly enriched in the AGE-RAGE signaling pathway. Molecular docking experiments confirmed strong binding interactions between RD’s primary active components (loureirin B and 7,4′-dihydroxyflavone) and RAGE’s extracellular domain, with binding energies below −5 kcal/mol, indicating high affinity. In vitro studies showed that RD (6.25 μg/mL) significantly suppressed AGEs-induced RAGE protein overexpression and downregulated inflammatory cytokine mRNA levels (IL-6, IL-1β, TNF-α), effectively alleviating chronic inflammation in diabetic wounds by blocking this pathway. The superior efficacy of the full RD extract over purified loureirin B in suppressing RAGE expression and oxidative stress strongly suggests synergistic interactions among its constituents. This is a critical observation, as it underscores the therapeutic advantage of using the multi-component botanical extract over isolated compounds, a phenomenon increasingly recognized in ethnopharmacology [54]. However, direct experimental evidence of such synergy is currently lacking, and future studies should include combination experiments to validate these potential synergistic effects.

Animal experiments further verified that the activation of the AGE-RAGE signaling pathway is associated with delayed diabetic wound healing. RD and its main component, LB, can significantly reduce the deposition of AGEs and RAGE on day 3 and day 5, accelerate the transition of the inflammatory phase, promote skin tissue regeneration in the late stage of wound healing, and thus speed up the wound healing process in diabetic mice. Macrophage polarization imbalance constitutes another critical factor in delayed diabetic wound healing [14]. Immunofluorescence revealed that RD hydrogel significantly reduced M1 macrophage (iNOS^+^CD68^+^) infiltration while promoting M2 macrophage (CD206^+^CD68^+^) polarization, particularly during the early inflammatory phase (days 3–5), thereby accelerating inflammation resolution and creating favorable conditions for subsequent healing phases. In contrast, while rb-bFGF (positive control) effectively promoted collagen deposition, it showed weaker macrophage polarization modulation, highlighting the superiority of RD in modulating the wound microenvironment during the inflammatory phase through macrophage polarization regulation.

However, our study has several limitations that should be acknowledged. First, the apparent discrepancy between the strong molecular docking affinity of 7,4′-DHF and its lack of cellular activity should be further investigated. Second, macrophage polarization was assessed using a limited set of markers (iNOS and CD206); future studies should include a broader panel to more comprehensively characterize phenotypic changes. Third, the absence of a fixation ring in the wound model may have facilitated contraction, potentially overestimating the intervention effects. Finally, systemic toxicity should be evaluated through histopathological examination of major organs. To address these limitations and facilitate clinical translation, future research should focus on standardizing extract quality, elucidating compound pharmacokinetics, and validating efficacy and safety through well-designed randomized controlled trials.

While our results support the therapeutic potential of RD, key practical issues must be addressed to advance its clinical translation. Drawing on the successful development of marketed natural product preparations (e.g., “Fespixon^®^” Xiangxue Tangzu Ointment for diabetic foot ulcers), our study provides a scientific foundation for the clinical application of RD hydrogel. Subsequent efforts should focus on three critical translational aspects: first, mitigating source variability through standardized quality control methods (e.g., HPLC) to ensure raw material consistency; second, investigating the topical bioavailability and local pharmacokinetic properties of key compounds (e.g., loureirins) to optimize delivery efficiency; and ultimately, validating the efficacy and safety of RD hydrogel through randomized controlled trials in diabetic patients. These studies represent essential steps toward achieving meaningful clinical translation.

## 5. Conclusions

Resina Draconis (RD) hydrogel promotes diabetic wound healing by regulating the AGE-RAGE signaling pathway, inhibiting oxidative stress and inflammatory responses, and promoting macrophage polarization toward the pro-healing M2 phenotype. While the complete RD extract demonstrated significant therapeutic efficacy, its main active component, loureirin B, showed inferior effects compared to the whole extract in terms of RAGE inhibition and inflammation alleviation. This suggests that other components in RD may synergistically enhance the overall therapeutic effects, though their specific mechanisms require further elucidation.

## Figures and Tables

**Table 1:** Primer sequences used for qPCR analysis (Mouse species).

Primer Name	Forward Sequence (5′-3′)	Reverse Sequence (5′-3′)
β-actin	CTATTGGCAACGAGCGGTTC	ACTGTGTTGGCATAGAGGTCTT
IL-1β	ATCTCGCAGCAGCACATCA	CCAGCAGGTTATCATCATCATCC
IL-6	TTCCATCCAGTTGCCTTCTTG	AATTAGCCTCCGACTTGTGAA
TNF-α	CCACGTCGTAGCAAACCACC	GTGAGGAGCACGTAGTCGG
COX-2	AGCCCATTGAACCTGGACTG	ACCCAATCAGCGTTTCTCGT
MCP-1	AGCCAACTCTCACTGAAGCC	GCGTTAACTGCATCTGGCTG

## Data Availability

The datasets generated and analyzed during this study are available from the corresponding author upon reasonable request. The RAW264.7 cell line used in this study is commercially available from Suzhou Haixing Biosciences Co., Ltd. (Catalog No: TCM-C677).

## Supplementary Materials

The following supporting information can be downloaded at https://www.mdpi.com/article/10.3390/cimb47090748/s1.

## Abbreviations

The following abbreviations are used in this manuscript:

RD	Resina Draconis
AGEs	Advanced Glycation End Products
RAGE	Receptor for Advanced Glycation End Products
LA	Loureirin A
LB	Loureirin B
LC	Loureirin C
LD	Loureirin D
7,4′-DHF	7,4′-Dihydroxyflavone
RSV	Resveratrol
CCK-8	Cell Counting Kit-8
STZ	Streptozotocin
rb-bFGF	Recombinant Human Basic Fibroblast Growth Factor
ROS	Reactive Oxygen Species
PPI	Protein–Protein Interaction
GO	Gene Ontology
KEGG	Kyoto Encyclopedia of Genes and Genomes
IL-1β	Interleukin-1β
IL-6	Interleukin-6
TNF-α	Tumor Necrosis Factor-α
COX-2	Cyclooxygenase-2
MCP-1	Monocyte Chemoattractant Protein-1

## References

[1] Luo Y.L., Luo W.J., Cao Y.N., Wang Z.P. (2025). m6A demethylase FTO/ALKBH5 promotes diabetes-induced endothelial cell dysfunction by negatively regulating lncRNA H19. Exp. Mol. Pathol..

[2] Zaidi S.M.A. (2016). Unani treatment and leech therapy saved the diabetic foot of a patient from amputation. Int. Wound J..

[3] Raghav A., Khan Z.A., Labala R.K., Ahmad J., Noor S., Mishra B.K. (2018). Financial burden of diabetic foot ulcers to world: A progressive topic to discuss always. Ther. Adv. Endocrinol..

[4] Guo S., DiPietro L.A. (2010). Factors Affecting Wound Healing. J. Dent. Res..

[5] Velnar T., Bailey T., Smrkoli V. (2009). The Wound Healing Process: An Overview of the Cellular and Molecular Mechanisms. J. Int. Med. Res..

[6] Koike S., Mitsuhashi H., Kishida A., Ogasawara Y. (2024). Elucidating the Antiglycation Effect of Creatine on Methylglyoxal-Induced Carbonyl Stress In Vitro. Int. J. Mol. Sci..

[7] Aronson D. (2008). Hyperglycemia and the pathobiology of diabetic complications. Adv. Cardiol..

[8] Kim W., Hudson B.I., Moser B., Guo J.C., Rong L.L., Lu Y., Qu W., Lalla E., Lerner S., Chen Y.L. (2005). Receptor for advanced glycation end products and its ligands—A journey from the complications of diabetes to its pathogenesis. Ann. N. Y. Acad. Sci..

[9] Reddy V.P., Aryal P., Darkwah E.K. (2022). Advanced Glycation End Products in Health and Disease. Microorganisms.

[10] Manigrasso M.B., Rabbani P., Egaña-Gorroño L., Quadri N., Frye L., Zhou B.Y., Reverdatto S., Ramirez L.S., Dansereau S., Pan J.H. (2021). Small-molecule antagonism of the interaction of the RAGE cytoplasmic domain with DIAPH1 reduces diabetic complications in mice. Sci. Transl. Med..

[11] Lin L. (2006). RAGE on the Toll Road?. Cell Mol. Immunol..

[12] González P., Lozano P., Ros G., Solano F. (2023). Hyperglycemia and Oxidative Stress: An Integral, Updated and Critical Overview of Their Metabolic Interconnections. Int. J. Mol. Sci..

[13] Wang J., Mao W., Yang Y., He F., Li J., Wang H.-H., Long J. (2024). Targeting the Receptor for Advanced Glycosylation End Products in Inflammation-Associated Diabetes Mellitus. J. Bio-X Res..

[14] Sun D., Chang Q., Lu F. (2024). Immunomodulation in diabetic wounds healing: The intersection of macrophage reprogramming and immunotherapeutic hydrogels. J. Tissue Eng..

[15] Zhou X., Guo Y.L., Xu C., Wang J. (2024). Macrophages: Key players in diabetic wound healing. World J. Diabetes.

[16] Qu M.Y., Jiang X., Zhou X.W., Wang O.R., Wu Q.Z., Ren L., Zhu J.X., Zhu S.S., Tebon P., Sun W.J. (2020). Stimuli-Responsive Delivery of Growth Factors for Tissue Engineering. Adv. Healthc. Mater..

[17] Shi Q.Y., Nong K.L., Vandvik P.O., Guyatt G.H., Schnell O., Rydén L., Marx N., Brosius F.I.I.I., Mustafa R.A., Agarwal A. (2023). Benefits and harms of drug treatment for type 2 diabetes: Systematic review and network meta-analysis of randomised controlled trials. Bmj-Brit Med. J..

[18] Baviera M., Foresta A., Colacioppo P., Macaluso G., Roncaglioni M.C., Tettamanti M., Fortino I., Genovese S., Caruso I., Giorgino F. (2022). Effectiveness and safety of GLP-1 receptor agonists versus SGLT-2 inhibitors in type 2 diabetes: An Italian cohort study. Cardiovasc. Diabetol..

[19] Norman G., Dumville J.C., Crosbie E.J. (2016). Antiseptics and Antibiotics for Surgical Wounds Healing by Secondary Intention Summary of a Cochrane Review. JAMA Dermatol..

[20] Schneider I., Calcagni M., Buschmann J. (2023). Adipose-derived stem cells applied in skin diseases, wound healing and skin defects: A review. Cytotherapy.

[21] Fan J.Y., Yi T., Sze-To C.M., Zhu L., Peng W.L., Zhang Y.Z., Zhao Z.Z., Chen H.B. (2014). A Systematic Review of the Botanical, Phytochemical and Pharmacological Profile of Dracaena cochinchinensis, a Plant Source of the Ethnomedicine “Dragon’s Blood”. Molecules.

[22] Liu Y., Zhao X.S., Yao R.Y., Li C.J., Zhang Z.L., Xu Y.H., Wei J.H. (2021). Dragon’s Blood from Worldwide: Species, Traditional Uses, Phytochemistry and Pharmacology. Am. J. Chin. Med..

[23] Al-Awthan Y.S., Bahattab O.S. (2021). Phytochemistry and Pharmacological Activities of *Dracaena cinnabari* Resin. Biomed. Res. Int.-UK.

[24] Gupta D., Bleakley B., Gupta R.K. (2008). Dragon’s blood: Botany, chemistry and therapeutic uses. J. Ethnopharmacol..

[25] Sun S.L., Wang P., Yue K.F., Yi Q.P., Xie X.J., Xie X.M. (2023). Effect and Mechanism of Dragon’s Blood on Wound Healing of Patients with Stress Hand Injury. Evid.-Based Compl. Alt. Med..

[26] Gao L.L., Liu T.M., Cheng X., Li N. (2020). Wound healing activity of a traditional Chinese medicine (Longxuejie) in capsule dosage form. Pak. J. Pharm. Sci..

[27] Fan W.J., Qu Y., Yuan X., Shi H.S., Liu G.B. (2024). Loureirin B Accelerates Diabetic Wound Healing by Promoting TGFβ/Smad-Dependent Macrophage M2 Polarization: A Concerted Analytical Approach Through Single-Cell RNA Sequencing and Experimental Verification. Phytother. Res..

[28] Ning S., Dai Z., Feng X. (2025). Chemical composition analysis and the determination of main components of Resina Draconis based on UPLC-QE-Orbitrap-MS. J. Shandong Univ. (Nat. Sci.).

[29] Li Y., Zang J., Wang X.M., Feng X.C., Qiu F. (2023). Deciphering the underlying wound healing mechanisms of (Lour.) Merr. by integrating network pharmacology, transcriptomics, and experimental validation. J. Ethnopharmacol..

[30] Sun J., Liu J.N., Fan B., Chen X.N., Pang D.R., Zheng J.A., Zhang Q.A., Zhao Y.F., Xiao W., Tu P.F. (2019). Phenolic constituents, pharmacological activities, quality control, and metabolism of species: A review. J. Ethnopharmacol..

[31] Xia T., Zhang M., Lei W., Yang R., Fu S., Fan Z., Yang Y., Zhang T. (2023). Advances in the role of STAT3 in macrophage polarization. Front. Immunol..

[32] Adams J.M., Cory S. (2007). Bcl-2-regulated apoptosis: Mechanism and therapeutic potential. Curr. Opin. Immunol..

[33] Zhou M., Liu X., Li Z., Huang Q., Li F., Li C.-Y. (2018). Caspase-3 regulates the migration, invasion and metastasis of colon ca ncer cells. Int. J. Cancer.

[34] Lee M.Y., Luciano A.K., Ackah E., Rodriguez-Vita J., Bancroft T.A., Eichmann A., Simons M., Kyriakides T.R., Morales-Ruiz M., Sessa W.C. (2014). Endothelial Akt1 mediates angiogenesis by phosphorylating multiple ang iogenic substrates. Proc. Natl. Acad. Sci. USA.

[35] Tito C., Masciarelli S., Colotti G., Fazi F. (2025). EGF receptor in organ development, tissue homeostasis and regeneration. J. Biomed. Sci..

[36] Leśniak W., Filipek A. (2023). S100A6 Protein—Expression and Function in Norm and Pathology. Int. J. Mol. Sci..

[37] Borgo C., D’Amore C., Sarno S., Salvi M., Ruzzene M. (2021). Protein kinase CK2: A potential therapeutic target for diverse human diseases. Signal Transduct. Target. Ther..

[38] Naba A. (2024). Mechanisms of assembly and remodelling of the extracellular matrix. Nat. Rev. Mol. Cell Biol..

[39] Hashimoto K., Higashiyama S., Asada H., Hashimura E., Kobayashi T., Sudo K., Nakagawa T., Damm D., Yoshikawa K., Taniguchi N. (1994). Heparin-binding epidermal growth factor-like growth factor is an autoc rine growth factor for human keratinocytes. J. Biol. Chem..

[40] Banfi C., Amadio P., Zarà M., Brioschi M., Sandrini L., Barbieri S.S. (2022). Prenylcysteine Oxidase 1 (PCYOX1), a New Player in Thrombosis. Int. J. Mol. Sci..

[41] Park J.-J., Lee S.J., Baek M., Lee O.-J., Nam S., Kim J., Kim J.Y., Shin E.-Y., Kim E.-G. (2024). FRMD6 determines the cell fate towards senescence: Involvement of the Hippo-YAP-CCN3 axis. Cell Death Differ..

[42] Nguyen D.-H.T., Gao L., Wong A., Chen C.S. (2017). Cdc42 regulates branching in angiogenic sprouting in vitro. Microcirculation.

[43] Sun J.S., Song L.Y., Zhou Y., Wu K.Y., Li C.Y., Han B.Q., Chang J. (2025). Review: Advances in multifunctional hydrogels based on carbohydrate polymer and protein in the treatment of diabetic wounds. Int. J. Biol. Macromol..

[44] Pianissoli L.G., Silva J.P.R., Penteado E.A., Lopes D.R.C., Mengal V.F. (2019). Oxidative Stress and Inflammation in Wound Repair: Molecular and Cellular Mechanisms. Faseb J..

[45] Chen X., Zhou X., Xia X., Chen Y., Mao T.C., Shi X.H., Zhang Y.M., Fan D.L. (2018). Retrospective analysis of related factors affecting skin wound healing. Int. J. Clin. Exp. Med..

[46] Eming S.A., Martin P., Tomic-Canic M. (2014). Wound repair and regeneration: Mechanisms, signaling, and translation. Sci. Transl. Med..

[47] Brem H., Tomic-Canic M. (2007). Cellular and molecular basis of wound healing in diabetes. J. Clin. Investig..

[48] Khanna S., Biswas S., Shang Y.L., Collard E., Azad A., Kauh C., Bhasker V., Gordillo G.M., Sen C.K., Roy S. (2010). Macrophage Dysfunction Impairs Resolution of Inflammation in the Wounds of Diabetic Mice. PLoS ONE.

[49] Chawla D., Bansal S., Banerjee B.D., Madhu S.V., Kalra O.P., Tripathi A.K. (2014). Role of advanced glycation end product (AGE)-induced receptor (RAGE) expression in diabetic vascular complications. Microvasc. Res..

[50] Mata R., Yao Y., Cao W., Ding J., Zhou T., Zhai Z., Gao C. (2021). The Dynamic Inflammatory Tissue Microenvironment: Signality and Disease Therapy by Biomaterials. Research.

[51] Hudson B.I., Lippman M.E. (2018). Targeting RAGE Signaling in Inflammatory Disease. Annu. Rev. Med..

[52] Kierdorf K., Fritz G. (2013). RAGE regulation and signaling in inflammation and beyond. J. Leukocyte Biol..

[53] Zhou M., Zhang Y., Shi L., Li L., Zhang D., Gong Z., Wu Q. (2024). Activation and modulation of the AGEs-RAGE axis: Implications for infl ammatory pathologies and therapeutic interventions—A review. Pharmacol. Res..

[54] Yang Y., Zhang Z., Li S., Ye X., Li X., He K. (2014). Synergy effects of herb extracts: Pharmacokinetics and pharmacodynamic basis. Fitoterapia.

