# Supplementary material for: Resina Draconis Promotes Diabetic Wound Healing by Regulating the AGE-RAGE Pathway to Modulate Macrophage Polarization
Source: Curr Issues Mol Biol. 2025 Sep 11;47(9):748. doi: 10.3390/cimb47090748 (PMC12468322; doi:10.3390/cimb47090748)
Supplement: Supplementary file 1 [file cimb-47-00748-s001.zip › cimb-3859399-supplementary.pdf]

Resina Draconis Promotes Diabetic Wound Healing by Regulating the AGE-RAGE Pathway to Modulate Macrophage Polarization

Xin Jin, Ang Li, Zhaoyuan Dai, Yi Li, Xinchi Feng * and Feng Qiu *

School of Chinese Materia Medica, Tianjin University of Traditional Chinese Medicine, 10 Poyang Lake Road, West Zone of Tuanbo New City, Jinghai District, Tianjin 301617, China; jinxin041@163.com (X.J.); lianghbkd@163.com (A.L.); zhaoyuandai1024@163.com (Z.D.); 15369620697@163.com (Y.L.)

* Correspondence: xiaochi0211@163.com (X.F.); fengqiu20070118@163.com (F.Q.)

Table S1. The chemical composition information of RD

Name	Molecular Formula	Molecular Weight
5-Hydroxy-3',4',7,8-tetramethoxyflavone	$C_{19}H_{18}O_7$	358.32
4'-Hydroxy-7-methoxy-8-methylflavan	$C_{17}H_{18}O_3$	270.32
7,4'-Dihydroxyflavone	$C_{15}H_{10}O_4$	254.24
7,4'-Dihydroxydihydroflavone	$C_{15}H_{12}O_4$	256.26
Apigenin	$C_{15}H_{10}O_5$	270.24
5,7,4'-Trihydroxy-8-methylflavone	$C_{16}H_{12}O_5$	284.26
7-Hydroxy-4'-methoxy-8-methylflavan	$C_{17}H_{18}O_3$	270.32
2'-Methoxy-4,4'-dihydroxychalcone	$C_{16}H_{14}O_4$	270.28
2,4,2',5'-Tetrahydroxydihydrochalcone	$C_{15}H_{14}O_5$	274.27
4',7-Dihydroxy-3'-methoxyflavan	$C_{16}H_{16}O_4$	272.30
7-Hydroxy-3'-methoxy-4'-butoxyflavone	$C_{22}H_{24}O_5$	368.42
8,4'-Dihydroxy-7-methoxy-6-methylflavan	$C_{17}H_{18}O_4$	286.32
2,6-Dimethoxy-4,4'-dihydroxydihydrochalcone	$C_{17}H_{20}O_5$	304.34
4'-Methoxy-3',7-dihydroxyflavone	$C_{16}H_{14}O_5$	286.28
7-Hydroxyflavone	$C_{15}H_{10}O_3$	238.24
7,4'-Dihydroxy-5-methoxy-8-methylflavone	$C_{16}H_{12}O_5$	284.26
3-Methylresveratrol	$C_{15}H_{14}O_3$	242.27
4',7-Dihydroxyflavan	$C_{15}H_{14}O_3$	242.27
5,7-Dihydroxy-4'-methoxyflavone	$C_{16}H_{12}O_5$	284.26
6,4'-Dihydroxy-7-methoxy-8-methylflavan	$C_{17}H_{18}O_4$	286.32
5,4'-Dihydroxy-7-methoxy-6-methylflavan	$C_{17}H_{18}O_4$	286.32
5,7-Dimethoxyflavone	$C_{17}H_{14}O_4$	282.29
5,7-Dimethoxyflavanone	$C_{17}H_{16}O_4$	284.31
7-Methoxydihydroflavone	$C_{16}H_{14}O_3$	254.28
Isoliquiritigenin	$C_{15}H_{12}O_4$	256.26
3,7,4'-Trihydroxy-5-methoxyhomoflavanone	$C_{16}H_{14}O_5$	286.28
5,7-Dihydroxy-2',4'-dimethoxydihydroisoflavone	$C_{17}H_{16}O_5$	300.31

4'-Methoxy-2,4-dihydroxychalcone	$C_{16}H_{14}O_4$	270.28
2,4,4'-Trihydroxychalcone	$C_{15}H_{12}O_4$	256.26
3,2',3',4'-Tetrahydroxy-4-methoxychalcone	$C_{16}H_{14}O_6$	302.28
2,4,4'-Trihydroxy-3'-methoxy-3-methyldihydrochalcone	$C_{17}H_{18}O_5$	302.32
4,4'-Dihydroxy-2,6-dimethoxydihydrochalcone	$C_{17}H_{18}O_5$	302.32
Loureirin D	$C_{20}H_{20}O_5$	340.37
Loureirin C	$C_{20}H_{20}O_4$	324.37
Loureirin A	$C_{16}H_{12}O_5$	284.26
Loureirin B	$C_{16}H_{12}O_5$	284.26
Cochinchinensin C	$C_{33}H_{34}O_7$	542.62
Cochinchinensin B	$C_{33}H_{34}O_6$	526.62
Cochinchinensin A	$C_{33}H_{36}O_6$	528.64
Resveratrol	$C_{14}H_{12}O_3$	228.24
p-Hydroxybenzoic acid	$C_7H_6O_3$	138.12
Pterostilbene	$C_{16}H_{16}O_3$	256.30

Table S2. Target information in the PPI network

Name	Degree	Betweenness Centrality	Closeness Centrality	Average Shortest Path Length	Clustering Coefficient	Neighborhood Connectivity	Radiality
TAT3	78	0.01161	1.00000	1.00000	0.67466	52.94872	1.00000
BCL2	77	0.01091	0.98734	1.01282	0.68079	53.19481	0.99984
CASP3	77	0.01091	0.98734	1.01282	0.68079	53.19481	0.99984
GAPDH	77	0.01091	0.98734	1.01282	0.68079	53.19481	0.99984
AKT1	75	0.01027	0.96296	1.03846	0.68685	53.44000	0.99951
EGFR	75	0.01051	0.96296	1.03846	0.68180	53.26667	0.99951
ESR1	74	0.00965	0.95122	1.05128	0.69234	53.63514	0.99934
HIF1A	74	0.00951	0.95122	1.05128	0.69456	53.70270	0.99934
MTOR	74	0.00968	0.95122	1.05128	0.69234	53.66216	0.99934
SRC	73	0.00931	0.93976	1.06410	0.69521	53.78082	0.99918
ALB	72	0.00880	0.92857	1.07692	0.69953	53.91667	0.99901
CCND1	72	0.00850	0.92857	1.07692	0.70657	54.16667	0.99901
HSP90AA1	72	0.00875	0.92857	1.07692	0.70031	54.02778	0.99901
TNF	71	0.00833	0.91765	1.08974	0.70463	54.12676	0.99885

BCL2L1	70	0.00779	0.90698	1.10256	0.71387	54.45714	0.99869
HSP90AB1	69	0.00780	0.89655	1.11538	0.70887	54.36232	0.99852
MMP9	69	0.00759	0.89655	1.11538	0.71270	54.42029	0.99852
GSK3B	65	0.00673	0.85714	1.16667	0.72212	54.95385	0.99786
PTGS2	63	0.00590	0.83871	1.19231	0.73477	55.22222	0.99753
JAK2	61	0.00511	0.82105	1.21795	0.74863	55.96721	0.99721
PIK3CA	61	0.00630	0.82105	1.21795	0.70273	54.00000	0.99721
RELA	61	0.00533	0.82105	1.21795	0.74098	55.54098	0.99721
RHOA	60	0.00534	0.81250	1.23077	0.73220	55.31667	0.99704
MAPK1	59	0.00498	0.80412	1.24359	0.74284	55.91525	0.99688
PARP1	59	0.00421	0.80412	1.24359	0.76914	56.30508	0.99688
CDC42	58	0.00525	0.79592	1.25641	0.72232	54.91379	0.99671
MCL1	58	0.00416	0.79592	1.25641	0.76407	56.55172	0.99671
IGF1R	57	0.00404	0.78788	1.26923	0.76441	56.87719	0.99655
MMP2	57	0.00392	0.78788	1.26923	0.77444	56.92982	0.99655
SIRT1	57	0.00403	0.78788	1.26923	0.76880	56.52632	0.99655
PPARG	56	0.00353	0.78000	1.28205	0.78247	57.05357	0.99638

KDR	55	0.00391	0.77228	1.29487	0.76364	56.63636	0.99622
MAPK8	53	0.00360	0.75728	1.32051	0.76633	56.94340	0.99589
TLR4	53	0.00301	0.75728	1.32051	0.79536	57.41509	0.99589
MAPK14	52	0.00286	0.75000	1.33333	0.79713	58.28846	0.99573
CXCR4	51	0.00255	0.74286	1.34615	0.80471	58.05882	0.99556
MAP2K1	51	0.00273	0.74286	1.34615	0.80235	58.35294	0.99556
AR	50	0.00267	0.73585	1.35897	0.79755	57.30000	0.99540
CDK4	50	0.00251	0.73585	1.35897	0.80653	57.86000	0.99540
BRAF	48	0.00220	0.72222	1.38462	0.81117	58.79167	0.99507
CCNB1	47	0.00154	0.71560	1.39744	0.85476	58.89362	0.99490
NFE2L2	46	0.00162	0.70909	1.41026	0.84638	59.54348	0.99474

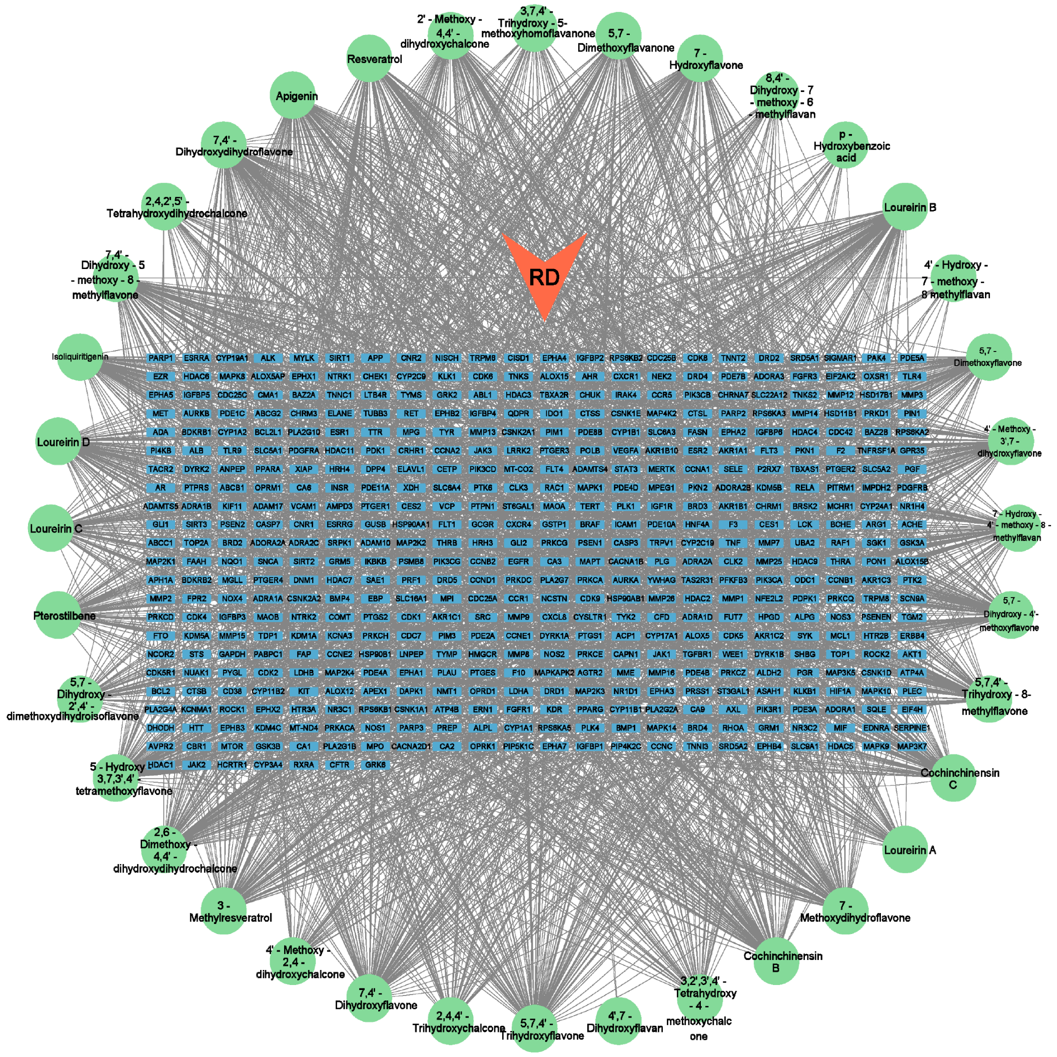

Figure S1. Visualization of the "compound-target-disease" network

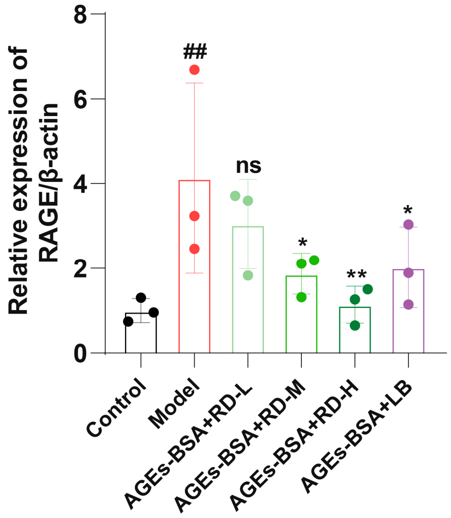

Figure S2. Quantification of RAGE mRNA expression levels by qPCR

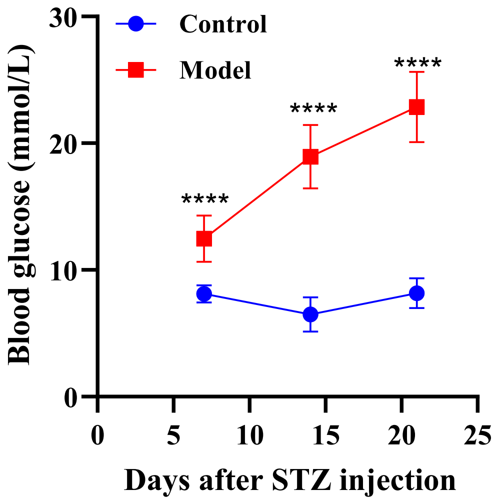

Figure S3. Blood glucose level in mice (Data are presented as mean ± SD). ****$p < 0.0001$ vs. Control group.

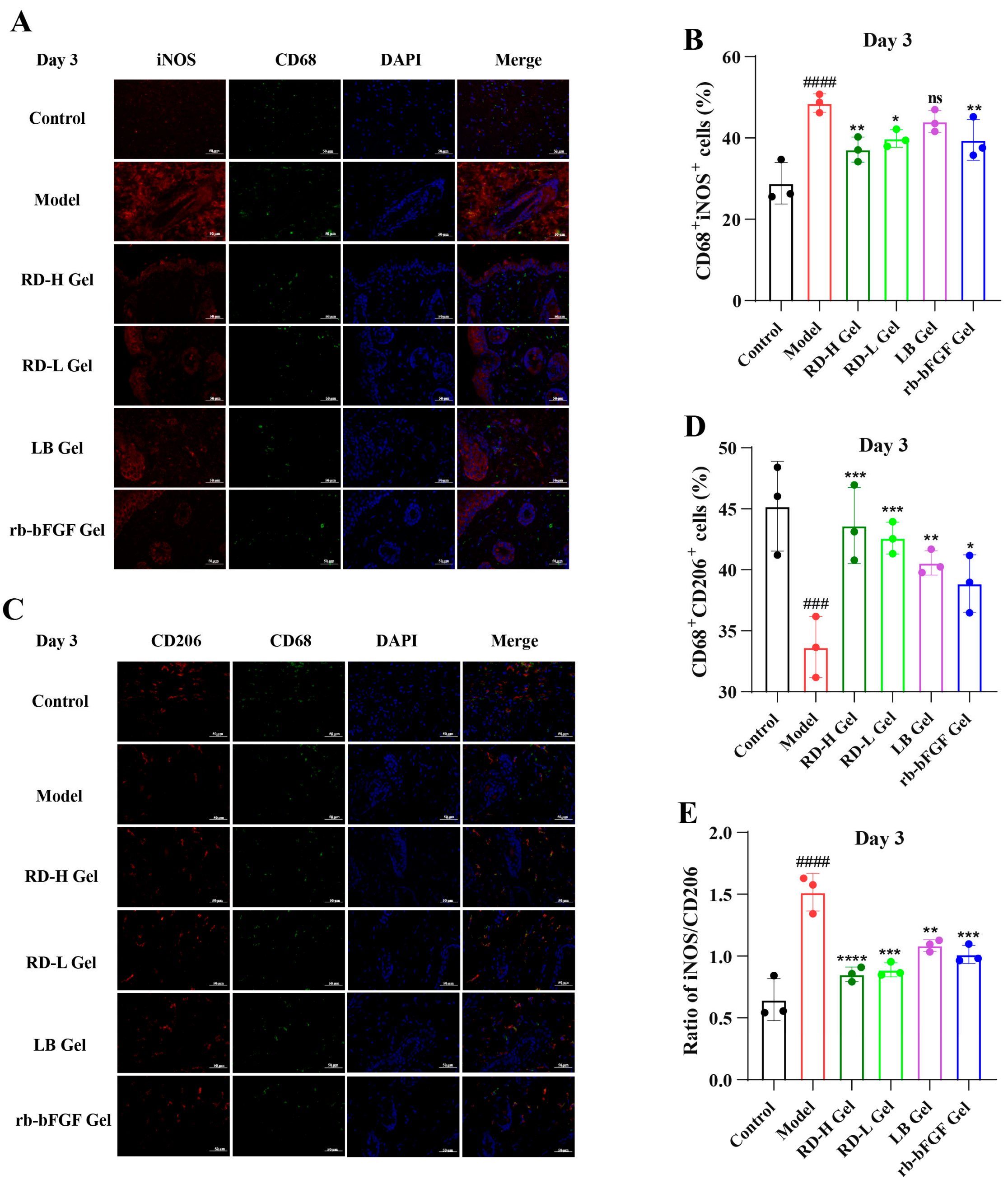

Figure S4. Evaluation of the regulatory effects of RD and LB on macrophage polarization in vivo. (A) Immunofluorescence staining of $iNOS^+$ (M1, red), $CD68^+$ macrophages (green), and DAPI (blue) at day 3 (Scale bar: 50 μm). (B) Semi-quantitative analysis of M1 macrophages ($CD68^+iNOS^+$ cells). (C) Immunofluorescence staining of $CD206^+$ (M2, red), $CD68^+$ macrophages (green), and DAPI (blue) at day 3 (Scale bar: 50 μm). (D) Semi-quantitative analysis of M2 macrophages ($CD68^+CD206^+$ cells). (E) Semi-quantitative analysis of the M1/M2 macrophage ratio. (For B, D, E: $n = 3$; data are presented as mean ± SD). $^{###}p < 0.001$, $^{####}p < 0.0001$ vs. Control group; $^{*}p < 0.05$, $^{**}p < 0.01$, $^{***}p < 0.001$, $^{****}p < 0.0001$ vs. model group; ns indicates no significant difference.
